# Lipidome Changes Associated with a Diet-Induced Reduction in Hepatic Fat among Adolescent Boys with Metabolic Dysfunction-Associated Steatotic Liver Disease

**DOI:** 10.3390/metabo14040191

**Published:** 2024-03-28

**Authors:** Helaina E. Huneault, Chih-Yu Chen, Catherine C. Cohen, Xueyun Liu, Zachery R. Jarrell, Zhulin He, Karla E. DeSantos, Jean A. Welsh, Kristal M. Maner-Smith, Eric A. Ortlund, Jeffrey B. Schwimmer, Miriam B. Vos

**Affiliations:** 1 Nutrition & Health Sciences Doctoral Program, Laney Graduate School, Emory University, Atlanta, GA 30322, USA; jwelsh1@emory.edu (J.A.W.); mvos@emory.edu (M.B.V.); 2 Department of Biochemistry, Emory School of Medicine, Emory University, Atlanta, GA 30329, USA; chih-yu.chen@emory.edu (C.-Y.C.); xueyun.liu@emory.edu (X.L.); eortlun@emory.edu (E.A.O.); 3 Section of Nutrition, Department of Pediatrics, School of Medicine, University of Colorado Anschutz Medical Campus, Aurora, CO 80045, USA; catherine.cohen@cuanschutz.edu (C.C.C.); kristal.m.maner-smith@emory.edu (K.M.M.-S.); 4 Division of Pulmonary, Allergy and Critical Care Medicine, Emory University, Atlanta, GA 30322, USA; zachery.ryan.jarrell@emory.edu; 5 Pediatric Biostatistics Core, Department of Pediatrics, School of Medicine, Emory University, Atlanta, GA 30322, USA; zhulin.he@emory.edu; 6 Division of Gastroenterology, Hepatology, and Nutrition, Department of Pediatrics, Emory University, Atlanta, GA 30322, USA; kdesant@emory.edu; 7 Children’s Healthcare of Atlanta, Atlanta, GA 30322, USA; 8 Department of Gastroenterology, Rady Children’s Hospital San Diego, San Diego, CA 92123, USA; jschwimmer@ucsd.edu; 9 Department of Pediatrics, School of Medicine, University of California, San Diego, CA 92093, USA

**Keywords:** lipidomics, phospholipids, triglycerides, metabolic-dysfunction-associated steatotic liver disease (MASLD), nonalcoholic fatty liver disease (NAFLD), insulin resistance, oxylipins

## Abstract

Little is known about lipid changes that occur in the setting of metabolic-dysfunction-associated steatotic liver disease (MASLD) regression. We previously reported improvements in hepatic steatosis, de novo lipogenesis (DNL), and metabolomic profiles associated with oxidative stress, inflammation, and selected lipid metabolism in 40 adolescent boys (11–16 y) with hepatic steatosis ≥5% (98% meeting the definition of MASLD). Participants were randomized to a low-free-sugar diet (LFSD) (n = 20) or usual diet (n = 20) for 8 weeks. Here, we employed untargeted/targeted lipidomics to examine lipid adaptations associated with the LFSD and improvement of hepatic steatosis. Our LC-MS/MS analysis revealed decreased triglycerides (TGs), diacylglycerols (DGs), cholesteryl esters (ChE), lysophosphatidylcholine (LPC), and phosphatidylcholine (PC) species with the diet intervention (*p* < 0.05). Network analysis demonstrated significantly lower levels of palmitate-enriched TG species post-intervention, mirroring the previously shown reduction in DNL in response to the LFSD. Targeted oxylipins analysis revealed a decrease in the abundance of 8-isoprostane and 14,15-DiHET and an increase in 8,9-DiHET (*p* < 0.05). Overall, we observed reductions in TGs, DGs, ChE, PC, and LPC species among participants in the LFSD group. These same lipids have been associated with MASLD progression; therefore, our findings may indicate normalization of key biological processes, including lipid metabolism, insulin resistance, and lipotoxicity. Additionally, our targeted oxylipins assay revealed novel changes in eicosanoids, suggesting improvements in oxidative stress. Future studies are needed to elucidate the mechanisms of these findings and prospects of these lipids as biomarkers of MASLD regression.

## 1. Introduction

Metabolic dysfunction-associated steatotic liver disease (MASLD) [1], formerly known as nonalcoholic fatty liver disease (NAFLD), is the most common liver disease in children [2,3]. Recently, a group of multinational liver societies announced that the term MASLD will replace NAFLD to provide an affirmative, non-stigmatizing framework for nomenclature and diagnosis. Under the new disease definition, MASLD includes the presence of at least one of five cardiometabolic risk factors in addition to hepatic steatosis (liver fat ≥ 5%) in the absence of alcohol consumption and other chronic liver diseases. For children and adolescents, these cardiometabolic risk factors include BMI ≥ 85th percentile for age/sex (BMI z-score ≥ +1), fasting serum glucose ≥ 100 mg/dL, blood pressure ≥ 130/80 mmHg, plasma triglycerides (TG) ≥ 100 mg/dL (age < 10 y) or ≥150 mg/dL (age ≥ 10 y), and plasma high-density lipoprotein (HDL)-cholesterol ≤ 40 mg/dL [1].

While previously rare, the prevalence of MASLD has risen markedly to an estimated 16.5% among all U.S. adolescents [4], including 26% among those with obesity [5,6]. Importantly, no recommended medications or supplements exist for treating pediatric MASLD beyond lifestyle modification for general weight loss, which has shown moderate success in reducing hepatic steatosis and serum alanine aminotransferase (ALT) [7,8,9,10,11,12,13].

Lipid dysregulation is a major factor in the onset and progression of MASLD [14]. Hepatic lipid accumulation results from an imbalance between free fatty acid acquisition and inadequate removal through beta-oxidation and very low-density lipoprotein (VLDL) secretion [15]. Excess free fatty acids in the liver can be converted to lipotoxic intermediates such as diacylglycerols (DGs) and ceramides (Cer) [16]. Lipotoxicity plays a pivotal role in the development of MASLD and the advancement to metabolic dysfunction-associated steatohepatitis (MASH) [1]. The buildup of toxic lipids within the hepatocytes ultimately leads to insulin resistance, oxidative stress, inflammation, and disease progression [17,18].

Experimental studies in children and adults have shown that high-sugar diets increase hepatic lipid accumulation through mechanisms such as increased de novo lipogenesis (DNL) [14,19,20,21,22]. We previously conducted a randomized controlled trial in 40 adolescent boys with hepatic steatosis ≥ 5%. A diet low in free sugars (restricted to <3% of daily caloric intake) for eight weeks resulted in a significantly greater reduction in hepatic steatosis (from 25% at baseline to 17% at week 8) compared to a usual diet (21% to 20%) [23], in parallel with a greater reduction in hepatic DNL [24]. To gain a deeper understanding of the biological changes associated with this diet treatment-induced reduction in hepatic steatosis, we performed high-resolution metabolomics and metagenomics on fasting plasma samples collected at baseline and study completion during the trial [25]. Our analysis revealed changes in key metabolic pathways, including several lipid pathways, such as omega-3 and linoleate metabolism, which may reflect improved oxidative stress and inflammation [25]. Additionally, previous studies comparing metabolome profiles of children with and without MASLD have also identified distinct differences in the presence of certain glycerophospholipids, including lysophosphatidylcholine (LPC) and lysophosphatidylethanolamine (LPE) species [26]. However, metabolomics has limitations for examining lipid species due to steps in the methodology that remove lipids.

We hypothesized that further insights into MASLD regression might be revealed through untargeted lipidomics, the large-scale study of lipid metabolism in biological systems [27,28]. Recent advances in lipidomics technology have uncovered distinct changes in lipid species related to MASLD onset and progression, including saturated fatty acids (SFA), TG, phospholipids, and Cer [29,30]. Additionally, a subset of lipid mediators known as oxylipins are involved in pro and anti-inflammatory pathways and have been linked with the progression of MASLD [31]. Oxylipins are derived from the enzymatic or non-enzymatic oxidation of membrane-bound polyunsaturated fatty acids (PUFAs), such as linoleic acid and arachidonic acid [32]. Enzymatic oxidation occurs via cyclooxygenases (COX), lipoxygenases (LOX), or cytochrome (CYP) P450s. Some of the most commonly studied oxylipins are eicosanoids, including prostaglandins, thromboxanes, and leukotrienes, which have a wide range of functions still being elucidated [33]. Recent studies in adolescents with MASLD have shown that plasma levels of certain pro-inflammatory oxylipins are associated with liver injury and inflammation [34,35].

While recent advancements have expanded our understanding of the onset and progression of pediatric MASLD [36,37], there is limited research on lipidome changes that occur with disease regression. To bridge this gap and build on our metabolomics analysis, this study aimed to investigate lipidome changes associated with reduced dietary sugar and hepatic steatosis in children with MASLD. To achieve this, we performed untargeted high-resolution lipidomics and targeted lipidomics on fasting plasma samples from 40 adolescent boys with steatosis who participated in the randomized, controlled LFSD intervention study described above [23].

## 2. Materials and Methods

### 2.1. Study Design

The parent study was a randomized, controlled dietary treatment trial conducted in 40 adolescent boys (ages 11–16 y) with magnetic resonance imaging-proton density fat fraction (MRI-PDFF)-proven hepatic steatosis [23]. The study was conducted between August 2015 and September 2017. As previously described, this study was performed solely with adolescent boys to ensure uniformity among participants, as MASLD predominantly affects males in the pediatric population [23]. Eligibility criteria included a clinical-pathological diagnosis of steatotic liver disease by liver biopsy (hepatic steatosis ≥ 5%). Additionally, evidence of active disease (hepatic fat > 10%, ALT > 45 U/L) and current sugar-sweetened beverage consumption (defined as ≥3, 8 fl oz drinks/week) was required for inclusion. Exclusion criteria included a history of diabetes or other chronic liver disease, history of significant alcohol use, or chronic use of medications known to cause hepatic steatosis or steatohepatitis. Both groups were asked not to make any major changes to their physical activity routines during the study. The LFSD intervention consisted of individualized menu planning and meal provision for the entire household to restrict free sugar intake to less than 3% of daily calories for eight weeks. The control group consumed their usual diet for eight weeks. The primary outcome of interest was a change in hepatic steatosis measured by MRI-PDFF from baseline to week eight. Fasting blood samples were collected at baseline and study completion to assess various laboratory markers, including liver enzymes (ALT, aspartate aminotransferase (AST), and gamma-glutamyl transferase (GGT)), blood glucose, insulin, and lipids. For the present study, homeostasis model assessment of insulin resistance (HOMA-IR) was calculated using the following formula: [fasting insulin (uIU/mL) × fasting glucose (mg/dL)/405] [38]. The triglyceride–glucose (TyG) index was calculated by applying the following equation: ln [fasting TG (mg/dL) × fasting glucose (mg/dL)/2] [39]. Blood samples were processed and stored in a freezer at −80 °C. Anthropometric assessments (height, weight, and waist circumference) were performed twice at each visit and averaged. To assess changes in dietary intake over the eight-week trial, three separate 24 h food recalls (two weekdays and one weekend day) were collected using the Nutrition Data System for Research (version 2015, University of Minnesota). Additionally, the NAFLD activity score (NAS) was calculated from each subject’s baseline histology report using the NASH Clinical Research Network scoring system [40]. A detailed summary of the parent study design, participant characteristics, and main findings for the effect of the dietary treatment on the primary and secondary outcomes of interest were previously reported [23]. Please see Table 1 below for a concise summary of the participant’s baseline characteristics.

**Figure 1:**
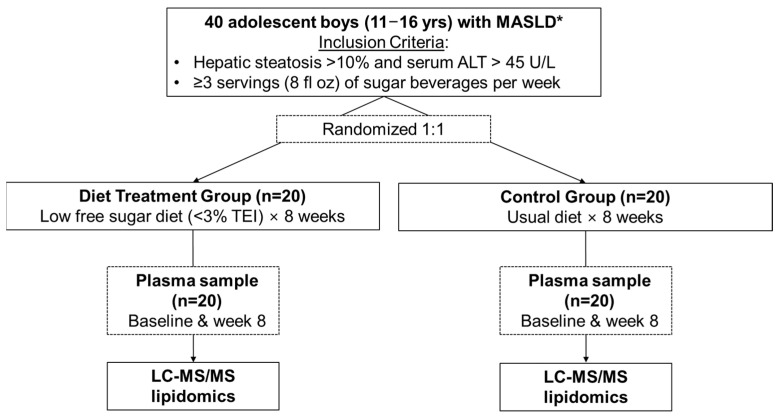
Workflow for participant inclusion in the present investigation. Lipidomics analysis was performed by liquid chromatography coupled with tandem mass spectrometry (LC-MS/MS). Abbreviations: ALT, alanine aminotransferase, TEI, total energy intake; * 98% of study subjects (39/40) met diagnostic criteria for MASLD, which includes the presence of at least one of the five cardiometabolic criteria in addition to hepatic steatosis ≥ 5%. The subject that did not meet MASLD diagnostic criteria was in the control group. Figure adapted from Cohen et al., 2023 [25].

In subsequent studies of data/samples from this parent trial, we reported the effects of the 8-week dietary treatment on hepatic DNL measured using a metabolic labeling protocol with heavy water [24] and on metabolomics and metagenomics profiles [25]. For the present study, which was designed as a follow-up study to build on these previous analyses, high-resolution lipidomics (both untargeted and targeted) were performed on stored plasma samples to assess the differential lipidome changes that occurred with the LFSD treatment. Figure 1 illustrates a summary of the workflow used for the present study. All study protocols were approved by the institutional review boards of the University of California San Diego and Emory University. Written informed consent was obtained from a parent or guardian, and assent was obtained from the adolescent participants.

### 2.2. Untargeted High-Resolution Lipidomics

Blood samples were drawn at baseline and week eight after an overnight fast. Plasma was collected in EDTA-coated tubes, processed immediately, and stored at −80 °C. High-resolution untargeted lipidomics were performed on stored plasma samples in August 2021, and targeted lipidomics were performed in August 2022 using established liquid chromatography–mass spectrometry (LC-MS/MS) methods [41,42]. To reduce the effect of freeze/thaw cycles on sample stability during processing, each sample was aliquoted with antioxidants (butylated hydroxytoluene (BHT)) before storage. Plasma samples were randomized before analysis, and lipids were extracted using a high throughput, monophasic, methyl t-butyl ether (MtBE)-based method. For this method, an automated pipetting and sample preparation system (Biotage Extrahera, Uppsala, Sweden) was used to load 50 µL of patient plasma into preconditioned wells containing 10 µL methanol and 10 µL of internal standard, Splash Lipidomix (Avanti Polar, Birmingham, AL, USA). To each well, 200 µL methanol containing 50 µg/mL BHT was then added, and the sample was mixed by three up-and-down passes of the automated sample handling pipette. The samples were centrifuged at 4000 rpm for five minutes to pellet precipitated protein. The supernatant was recovered and transferred to a separate 96-deep well plate. To extract lipids from the supernatant, 250 µL MtBE:methanol (3:1 *v*/*v*) was added to all wells and mixed with three up and down passes of the automated sample handling pipette. The sample plate was then centrifuged at 1000 *g* for three minutes, and the supernatant was filtered through a 0.25 µm polytetrafluoroethylene (PFTE) filter plate (Biotage, ISOLUTE^®^ FILTER+ (Uppsala, Sweden). The recovered extract was then dried under nitrogen gas and subsequently reconstituted to 200 µL in acetonitrile: isopropanol (1:1 *v*/*v*) methanol. Extracted plasma samples were then resolved on a Vanquish UHPLC (Thermo Scientific, Waltham, MA, USA) using a Thermo Scientific Accucore C_18_ (4.6 × 100 mm, 2.6 µm) column on a 15 min linear gradient, whereby Solvent A was 60:40 acetonitrile: water and Solvent B 90:10 isopropanol:acetonitrile. Both solvents in the mobile phase contained 0.1% formic acid and 10 mM ammonium formate. The column temperature was set at 50 °C and a constant flow rate of 0.4 mL/min. All untargeted chromatography parameters are shown in Table S1. Next, eluted lipids were analyzed by a Thermo IDX Fusion mass spectrometer operated in both the negative and positive ionization modes (Thermo Scientific, Waltham, MA, USA). A data-dependent acquisition method was used to complete the untargeted lipidomics analysis. For this method, a high-resolution MS scan was conducted on each sample at 120,000 FWHM resolution, and ions above the instrumental noise threshold were systematically fragmented for structural elucidation. All MS/MS spectra were conducted using 30,000 FWHM resolution. For quality control, a pooled sample was prepared and run after every 10 samples, and the analytical signal of the internal standard was monitored to ensure spray stability and injection volume. All instrumental parameters used for untargeted lipidomics are shown in Table S2, and all the validated lipid species are listed in Table S3.

### 2.3. Targeted Lipidomics (Oxylipins Assay)

To further characterize downstream changes in polyunsaturated fatty acid (PUFA) metabolism, including linoleate and arachidonic acid, we performed a targeted oxylipin assay to identify and quantify relevant oxylipins and endocannabinoids in plasma samples as previously described [43,44,45,46]. Briefly, an automated C_18_ solid phase extraction (SPE) manifold, the Biotage Extrahera (Biotage, Uppsala, Sweden), was used for plasma sample extraction. The C_18_ cassettes were conditioned using ethyl acetate, methanol, and water. Next, 100 µL of patient plasma was spiked with 1% BHT solution to a final BHT concentration of 0.1% and pH of 3.0 by acetic acid addition. Then, each sample was deposited on the matrix and rinsed with three column volumes of water and hexane. The fraction containing endocannabinoids and oxylipins was eluted off the SPE column with 400 µL methyl formate. The recovered oxy/endo fraction was then dried under nitrogen gas and reconstituted in 100 µL of methanol before chromatography. Oxylipins were analyzed using an Agilent 1290 Infinity II/6495c (Agilent, Santa Clara, CA, USA) LC-MS/MS system, whereby a 10 µL sample was injected onto a Thermo Accucore C_18_ column (100 × 4.6, Thermo, Waltham, MA, USA) and resolved on a 16 min gradient using water as Solvent A and acetonitrile as Solvent B; both contained 10 mM ammonium acetate. The column was heated to 50 °C in a temperature-controlled column chamber, and a 0.5 mL/min flow rate was used for analysis. Eluted oxylipins and endocannabinoids were analyzed by the online Agilent 6495c (Agilent, Santa Clara, CA, USA) mass spectrometer operated in both negative and positive ionization modes. Instrumental parameters were optimized using external analytical grade standards and were held consistent throughout the course of the analysis. Additionally, a pooled sample for quality control was placed every 10 samples throughout the plate and at the beginning and end of the run. Oxylipins were analyzed in negative ion mode, while endocannabinoids were analyzed in the positive ion mode using the instrument’s rapid polarity switching feature (<25 ms capability). Both classes of lipids were targeted using a multiple reaction monitored (MRM) based method whereby detected lipids were fragmented and quantified against external standard curves. Please see Tables S4–S6 for chromatography and instrumental parameters and the oxylipin transitions used.

### 2.4. Untargeted Lipidomic Data Processing

LipidSearch software version 4.2 (Thermo Fisher Scientific, San Jose, CA, USA) was used to process the raw data obtained from HPLC-MS/MS analysis. The procedures performed by the software covered peak identification, lipid identification, peak extraction, peak alignment, and quantification. The software settings included precursor tolerance, 5 ppm; product tolerance, 5 ppm; and product ion threshold, 5%. The result was a 2-dimensional “feature table” consisting of 273 C_18_/ESI+ high confidence identifications defined by an accurate mass-to-charge ratio (*m*/*z*) and retention time (RT). Ion abundance in each sample was MS/MS verified. Approximately 76% of the data had a coefficient of variation (CV) < 30% with a signal-to-noise ratio greater than three. The positive ion mode dataset was normalized by class specific internal standards. Missing values were imputed with half the feature minimum, and there were no features with missing values > 20%. To achieve normal data distribution, samples were log_2_ transformed, auto-scaled, and median-centered using R version 4.2.3 [47].

### 2.5. Targeted Oxylipins Data Processing

To quantify oxylipins and endocannabinoids, the respective MRM transition list was uploaded to Skyline version 21.2 (University of Washington, Department of Genome Sciences) to process the raw data for peak identification and peak area integration. The peak area was calibrated against corresponding external standards, and the output was exported to R version 4.2.3 for further data normalization. The concentration CV and retention time (RT) CV were calculated using pooled QC samples. Analytes were removed if the concentration CV was ≥65% or the RT CV was >10%. Additionally, analytes with missing values > 20% across all groups were removed; otherwise, missing values were imputed with half the feature minimum. To achieve a normal distribution of the data, samples were log_2_ transformed, auto-scaled, and median-centered prior to statistical analysis.

### 2.6. Statistical Analyses

#### 2.6.1. Univariate Analysis

To examine changes in lipid class profiles, the sum of all lipid species within the same lipid class and lipid class ratios were calculated using the normalized dataset. The difference in means between baseline and week eight was assessed using a paired sample *t*-test, and the difference in means between baseline values of different groups was assessed using students *t*-test. *p*-values < 0.05 were considered statistically significant. Results were visually presented using boxplots.

#### 2.6.2. Multivariate Analysis

To examine variations in the lipidome from baseline to week 8 within the dietary intervention group, multivariate analysis was applied using the R package mixOmics [48]. The R package mdatools [49] was used for cross-validation and calibration. First, unsupervised principal component analysis (PCA) was implemented, followed by supervised partial least squares discriminant analysis (PLS-DA) to visually examine if separation of the lipidome occurred with the diet treatment. Additionally, variable importance in projection (VIP) scores from the analysis were calculated using the mdatools::vipscores function, and features with the top 20 VIP scores at 3 components were identified [49].

#### 2.6.3. Pairwise Differential Expression Analysis

Pairwise differential expression (DE) analysis was performed using both the untargeted and targeted lipidomics datasets. To identify individual lipids that changed with the dietary treatment, we used the duplicateCorrelation() function to calculate the correlation between repeated samples. Then, we added a blocking variable in a linear mean-reference model using the limma::lmFit function. Limma with empirical Bayes variance shrinkage (limma::eBayes) was then used for DE analysis. The log fold change versus the statistical significance (*p* < 0.05) was visually presented using volcano plots. To gain a deeper understanding of the heterogeneity in response to the LFSD among the adolescents with MASLD, we stratified participants in the intervention group into two subgroups: responders (participants who demonstrated a decrease in hepatic steatosis of ≥5%) and non-responders (participants who demonstrated < 5% decrease or an increase in hepatic steatosis with the dietary intervention). The differences in baseline clinical variables and lipid features between responders and non-responders were analyzed using DE analysis, and the relative fold changes were compared in response to the LFSD. Analytical and data visualization tools were performed using limma, ggrepel, stringi, and ggplot2 R packages [50,51,52].

#### 2.6.4. Correlation Analysis of Lipids with Hepatic Steatosis and DNL

We used the R package rmcorr [53] to calculate repeated measures correlations between individual lipid features with hepatic steatosis and DNL. Additionally, heatmaps were created using the R packages ComplexHeatmap [54] and pheatmap [55].

#### 2.6.5. Weighted Lipid Co-Expression Network Analysis

To find clusters (modules) of highly correlated lipids, a weighted lipid co-expression network was built with the WGCNA R package as previously described [56]. Briefly, a total of 40 participants—20 in the intervention group and 20 in the control group—were included in the analysis, contributing 40 measurements from baseline and 40 measurements from Week 8. One outlier from the dietary intervention group at Week 8 was identified in the sample clustering analysis and was removed before our WGCNA. The findings from the dietary intervention group are reported in our Section 3. A soft threshold with a power of 7 was chosen (scale-free fit index, R^2^ = 0.942), and the assigned network was generated by the component-wise average values for topologic overlap (TO). Lipid features were hierarchically clustered by distance measured using dissTom (1-TO). Additionally, the WGCNA::blockwiseModules() function was used to determine the lipid modules (Arguments: corType = bicor, mergecutHeight = 0.20, deepSplit = 2, and minModules = 7; the default setting for others). The majority of the default parameters of the WGCNA R package were implemented; however, the manual settings outlined above were selected based on our dataset. After network analysis, correlations between clinical traits and the identified modules as well as the module eigenlipid values (MEs; the first principal component of a given module) were assessed using a repeated measures correlation analysis for each subject. The network was plotted using the R packages igraph [57] and ggraph [58]. Eigenvector centrality was calculated to quantify the influence of a node (module) within the networks.

#### 2.6.6. Over-Representation Analysis

To identify the lipid classes that were enriched within each WGCNA module, an over-representation analysis was performed using one-sided Fisher exact *t*-tests with all annotated lipids as the background. Hub lipids (module membership (MM) > 0.65) within each module were analyzed as the query. Raw *p*-values were adjusted within each module using the Benjamini–Hochberg procedure, and significantly enriched lipid classes were selected at a 5% false discovery rate (FDR).

#### 2.6.7. Lipid Set Enrichment Analysis (LSEA)

LSEA, a ranked-based enrichment analysis using an algorithm for gene set enrichment analysis (GSEA) [59], was applied to the untargeted normalized dataset, and the DE analysis results at the level of individual lipids were used as input for ranking metric values. LSEA determines whether any a priori-defined lipid classes are overrepresented at a ranked list’s extremes (top or bottom). The R package fgsea [60] was used for analysis, and the minimal size of a lipid class was set at 2. A total of 10,000 permutations were used to estimate enrichment *p*-values and to calculate normalized enrichment scores (NES). Raw *p*-values were adjusted within each contrast using Benjamini–Hochberg procedures, and significantly enriched lipid classes were selected at a 5% FDR. The hub lipid species (MM > 0.65) in each module were selected from the WGCNA and then used for LSEA.

#### 2.6.8. Dietary Fat Intake Analysis

The NDSR dietary reports from a sub-sample of 20 participants from Emory University were analyzed using R version 4.2.3. Paired *t*-tests were used to evaluate changes in the intake of dietary fats from baseline to week eight. Statistical significance was set at *p* < 0.05. Missing data was imputed using the respective median value of the dietary variable from participants in the intervention (n = 10) or control group (n = 10).

## 3. Results

In addition to hepatic steatosis (liver fat ≥ 5%), 98% of study subjects (39/40) met diagnostic criteria for MASLD, which includes the presence of at least one of five cardiometabolic criteria defined above [1]. The subject that did not meet MASLD diagnostic criteria was in the control group. Most participants (95%) were of Hispanic race/ethnicity with a mean age of 13.0 ± 1.9 years. Dietary free sugar intake was similar in the intervention and control group at baseline (10% total energy intake (TEI) in the diet treatment group vs. 11% TEI in the control group) but decreased to <1% TEI at week eight in the diet treatment group, compared to 10% TEI at week eight in the control group. As previously reported, the diet treatment group also experienced a greater decrease in hepatic steatosis measured by MRI-PDFF (from 25% to 17%) compared with the control group (from 21% to 20%), as well as ALT, AST, GGT, and total cholesterol (all *p* < 0.05) [23]. Hepatic DNL was significantly decreased in the treatment group (from 34.6% to 24.1%) versus the control group (33.9% to 34.6%) (adjusted week eight mean difference: −10.6% (95% CI: −19.1%, −2.0%)) and body weight decreased slightly (from 91.1 to 89.7 kg) in the treatment group [24]. Furthermore, our previous metabolomics and metagenomics analysis revealed differential changes in the microbiome and metabolome, including amino acid and lipid species associated with the diet treatment and improvement in hepatic steatosis [25].

### 3.1. Changes in Dietary Fat Intake and Fatty Acid Composition

As published in our previous study, total dietary fat intake was similar in both groups at baseline (32% TEI in the diet treatment group vs. 33% TEI in the control group). This increased to 36% TEI at week eight in the diet treatment group, while the control group remained at 33% TEI [21]. In the present study, a more detailed analysis of changes in dietary fats was conducted in a sub-sample of participants from the Emory University study site (n = 20). Among participants in the intervention group (n = 10), there were no significant changes in total dietary fats or fatty acid intakes from baseline to week 8. In the control group (n = 10), total cholesterol intake significantly increased (*p* = 0.017; Table S7), while other dietary fats did not show significant changes. 

### 3.2. Lipidome Changes Associated with the LFSD Treatment

Our untargeted lipidomics analysis identified 273 distinct lipid features in the plasma samples, which were included in univariate and multivariate data analyses. Investigation of the lipid features revealed minimal differences between the control group at baseline compared to week 8 (Figure S1A–D). Therefore, we chose to focus on changes that occurred in the diet treatment group going forward.

**Figure 2:**
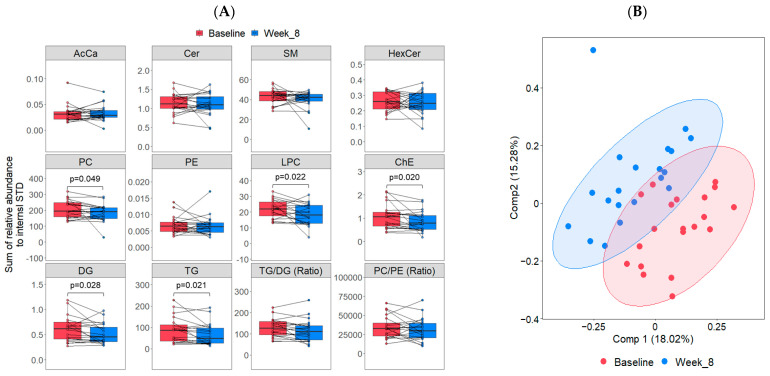

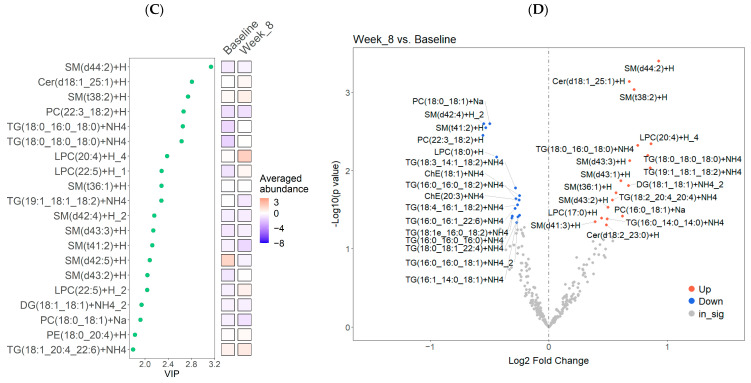
Identification of differentially abundant lipids in the diet treatment group (n = 20) at baseline compared to Week 8 (post-intervention). (**A**) Boxplots illustrating all matched paired measurements (baseline and Week 8) for each lipid class, including the median and interquartiles. Paired sample *t*-tests were used to identify significant differences (*p* < 0.05) between the means of the matched pairs. Statistically significant *p*-values are shown in the figure. (**B**) Supervised PLS-DA analysis of lipidomic data. The first two latent components (Comp 1 and Comp 2) are presented with 95% confidence ellipses surrounding the cluster core. (**C**) VIP plot illustrating the average normalized values of the top 20 VIP scores from the PLS-DA analysis. (**D**) Volcano plot showing the log_2_ fold change versus the −log_10_(*p*-value) for pairwise comparisons. Lipid species that significantly increased in abundance are shown in red, while those that significantly decreased are indicated in blue.

Of the 10 lipid classes measured among participants in the intervention group, the first- and second-highest in net abundance were plasma triglycerides (TG) (43.96%) and sphingomyelins (SM) (23.44%), respectively (Figure S1E). Univariate analysis of the changes in lipid classes that occurred with the diet treatment revealed a significant decrease in total cholesteryl esters (ChE), TGs, diacylglycerols (DGs), lysophosphatidylcholine (LPC), and phosphatidylcholine (PC) species from baseline to week 8 (all *p* < 0.05; Figure 2A). The TG/DG ratio also decreased (*p* = 0.05; Figure 2A). Unsupervised PCA showed no apparent separation between baseline and week eight (Figure S1F). However, the PLS-DA revealed slight separation of the lipidome after the diet treatment (Figure 2B), and the cross-validation results showed that the prediction accuracy between groups was 0.6 with an optimal number of 3 components. Variable importance in projection (VIP) analysis was used to identify the most discriminant lipids characterizing baseline from Week 8 within the diet treatment group, which included several SM, phospholipid, and TG species (Figure 2C). Furthermore, the DE analysis results (Table S8), illustrated as a volcano plot (Figure 2D), revealed that the diet treatment was associated with a significantly altered abundance of 34 lipid features (*p* < 0.05). This included an increase in the abundance of several SM and TG species, two LPC species, two Cer species, one PC species, and one DAG. There was also a decrease in the abundance of several TGs, two PC species, two ChE, two SM species, and one LPC species.

### 3.3. Correlations between Hepatic Steatosis and DNL with the Untargeted Lipidomics Data 

**Figure 3:**
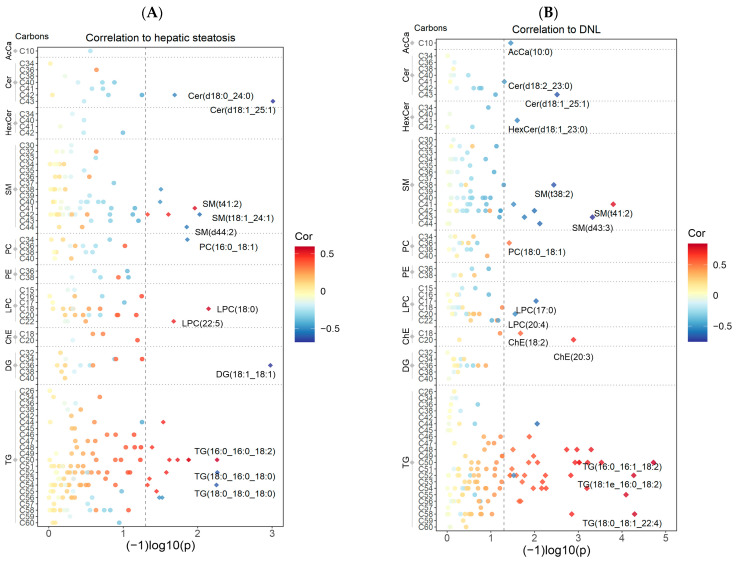
Repeated measures correlations between individual lipid species, hepatic steatosis, and DNL among participants in the diet treatment group (n = 16–19). The acyl chain length sorted by lipid class is shown on the y-axis and −log_10_ (*p*-value) on the x-axis. Correlations are presented on a color scale from −1 (blue) to 1 (red). The dashed line indicates a *p*-value cutoff of <0.05. Lipid species with a significant correlation to hepatic steatosis (**A**) or DNL (**B**) are indicated with a diamond shape (◊), and the top correlates in each lipid class are labeled. One participant was missing MRI data for Week 8, and four participants were missing DNL data. Therefore, 19 and 16 participants were included in the hepatic steatosis and DNL correlation analyses, respectively.

Among the 273 individual lipid species from 10 lipid classes, 28 species showed significant associations with hepatic steatosis in the diet treatment group (Figure 3A). The top lipid species that reached statistical significance were Cer(d18:1_25:1) (cor = −0.68, *p* = 9.7 × 10^−4^, DG(18:1_18:1) (cor = −0.68, *p* = 1.1 × 10^−3^), SM(t18:1_24:1) (cor = −0.57, *p* = 9.4 × 10^−3^), LPC(18:0) (cor = 0.58, *p* = 7.1 × 10^−3^), TG(18:0_16:0_18:0) (cor = −0.60, *p* = 5.4 × 10^−3^), PC(16:0_18:1) (cor = −0.54, *p* = 0.013). Additionally, a total of 51 lipid species showed significant associations with DNL (Figure 3B). The top lipid species reaching statistical significance were AcCa(10:0) (cor = −0.51, *p* = 0.035), Cer(d18:1_25:1) (cor = −0.67, *p* = 0.003), HexCer(d18:1_23:0) (cor = −0.54, *p* = 0.025), SM(t41:2) (cor = 0.79, *p* = 1.6 × 10^−4^), LPC(17:0) (cor = −0.61, *p* = 0.009), PC(18:0_18:1) (cor = 0.51, *p* = 0.038), ChE(20:3) (cor = 0.71, *p* = 1.3 × 10^−3^), TG(16:0_16:1_18:2) (cor = 0.85, *p* = 1.9 × 10^−5^). Notably, Cer(d18:1_25:1) and SM(t41:2) showed the most prominent correlations with both hepatic steatosis and DNL. Please see Tables S9 and S10 for all correlations, *p*-values, and 95% confidence intervals. Additionally, please see Figure S2, which illustrates the individual lipid species with the top positive and negative correlations with hepatic steatosis and DNL.

### 3.4. Construction of a Lipid Coexpression Network

**Figure 4:**
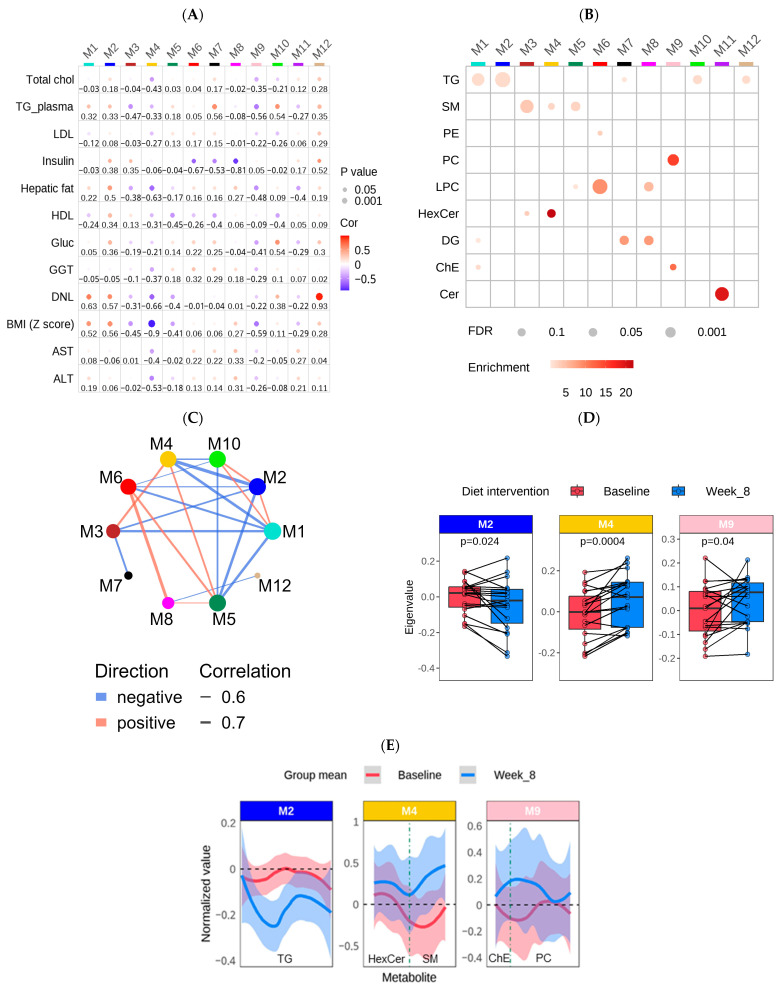
Network analysis of untargeted lipidomics and clinical data using WGCNA among participants in the diet treatment group (n = 39 samples (20 baseline & 19 week 8; one outlier was removed at week 8)). (**A**) A bubble heatmap showing the correlations between the lipid modules (M1–12) and clinical traits using a color scale from −1 (blue) to 1 (red). The size of the bubble indicates the *p*-value (the larger the bubble size, the lower the *p*-value), and the number indicates the correlation coefficient. (**B**) A bubble heatmap showing the lipid class enrichment in each of the 12 modules. The size of the bubble indicates the *p*-value (the larger the bubble size, the lower the *p*-value), and the color of the bubble indicates the overlap (enrichment) of the lipid classes in each module. *p*-values were calculated using Fisher’s exact test. (**C**) The correlations between each module in the network analysis. The correlation cutoff was 0.5 and *p* < 0.05. (**D**) Boxplots showing the difference in ME values between pre- (baseline) and post- (Week 8) dietary intervention. *p*-values achieving statistical significance (*p* < 0.05) are presented in the figure. (**E**) Line charts with 95% confidence intervals highlighting the average change in normalized values from baseline to Week 8 for the blue, pink, and yellow modules. Each module’s hub lipid classes (MM > 0.65) are indicated at the bottom of each plot and are separated by a dashed line. The order of the lipids is based on the chain length.

To identify groups (modules) of lipids significantly associated with clinical traits, the normalized dataset of 273 lipids was used to generate a lipid co-expression network with the WGCNA algorithm. As depicted in Figure 4A,B, the resulting network consisted of 12 lipid modules ranging in size from 7 to 43 lipids. Detailed information—including module membership (MM), module epigenlipid values (MEs), *p*-values, and other module information—are shown in Figure S3 and Tables S11–S13. To assess whether a given co-expression network module was related to clinical traits, we correlated the MEs to ALT, AST, GGT, HDL, LDL, total cholesterol, fasting glucose, insulin, hepatic steatosis, plasma TG, BMI z-scores, and DNL (Figure 4A). The lipid classes in which each module is enriched are shown in Figure 4B. Based on the WGCNA results, 5 out of 12 modules were enriched in TG, and except for Module 7 (M7; black), M1 (turquoise), M2 (blue), M12 (tan), and M10 (greenyellow) were highly related to each other (Figure 4C). Notably, M12 (tan), M1 (turquoise), and M2 (blue) were significantly correlated with DNL (M12 (tan): cor = 0.93, *p* = 2.78 × 10^−7^; M1 (turquoise): cor = 0.63, *p* = 9.15 × 10^−3^; M2 (blue): cor = 0.58, *p* = 1.98 × 10^−2^). M12 (tan) demonstrated the strongest correlation with DNL and was predominantly composed of ether-containing TG (Figure 4A,B; Table S11). Interestingly, approximately 71% of the TG species in M2 (blue) contained palmitate (16:0), while only 28% of the TG species contained palmitate in M1 (turquoise) (Figure S4A, Table S11). Additionally, M7 (black) was significantly enriched in DG, and M10 (greenyellow) contained only TG. Both modules were significantly associated with plasma TG (M7 (black): cor = 0.56, *p* = 0.011: M10 (greenyellow): cor = 0.54, *p* = 0.014). Compared to M7 (black), M8 (magenta) was primarily enriched in DG of higher unsaturation (Figure 4B and Figure S4B, Table S11) and was not correlated with plasma TG (M8 (magenta): cor = −0.08, *p* = 0.73). Furthermore, both M8 (magenta) and M6 (red) were enriched in LPC (primarily LPC (16:0), LPC (18:0), LPC (18:1), and LPC (18:2)), and were negatively correlated to fasting insulin levels (M8 (magenta): cor = −0.81, *p* = 0.0076; M6 (red): −0.67, 0.0497). M10 (greenyellow) was the only module significantly related to plasma glucose levels (cor = 0.54, *p* = 0.0135), and all of the TG species in this module contained oleic acid (18:1) as well as fatty acids with >20 carbons. M3 (brown) and M5 (green), which were enriched in SM, were negatively correlated to plasma TG and HDL levels, respectively (Table S13). Lastly, M11 (purple) was significantly enriched in ceramide species and was negatively correlated with hepatic steatosis. 

Importantly, the modules that significantly changed with the dietary intervention were M4 (yellow) and M9 (pink), which increased in abundance, and M2 (blue), which decreased in abundance (Figure 4D,E). The hub lipid classes (MM > 0.65) in M4 (yellow) were SM and HexCer (primarily very-long-chain SM(d40:1), SM(d43:1)) and Hex2Cer(d18:1_24:1)), and those in M9 (pink) were PC (primarily PC(36:4), PC(16:0_20:4), PC(18:0_20:4)), and ChE (Table S11). However, only the PC in M9 (pink) reached statistical significance in the LSEA (*p* = 6.5 × 10^−5^). Both modules were negatively correlated to hepatic steatosis (M4 (yellow): cor = −0.63, *p* = 0.004; M9 (pink): cor = −0.48, *p* = 0.037). Additionally, the lipids in M2 (blue), which primarily contained palmitate-enriched TG, as mentioned above, were positively correlated with hepatic steatosis (cor = 0.50, *p* = 0.031) as well as DNL (cor = 0.58, *p* = 0.020). Of note is that the lipids in M12 (tan), which showed the highest correlation with DNL (cor = −0.93, *p* = 2.78 × 10^−7^), also decreased in abundance after the dietary intervention. However, the change in ME between baseline and week 8 was non-significant (Figure S4C, Table S13).

### 3.5. Oxylipin Changes Associated with the LFSD Treatment

Among participants in the intervention group, the DE analysis revealed a significant decrease in the abundance of 8-isoprostane and 14,15-dihydroxy-5Z,8Z,11Z-eicosatrienoic acid (14,15-DiHET) along with a significant increase in 8,9-dihydroxy-5Z,11Z,14Z-eicosatrienoic acid (8,9-DiHET) after the LFSD (all *p* < 0.05, Figure S5A). Additionally, total DiHETs and total DiHETs plus total EETs significantly increased after the diet treatment (*p* < 0.05). In contrast, total EETs and the ratio of DiHET/EET did not significantly change in our study (Figure S5B).

### 3.6. Subgroup Analysis: Untargeted Lipidomic Data

In order to take into account individual response, we separated participants in the intervention group into responders (reduction in hepatic steatosis of ≥5% post-intervention) and non-responders (<5% reduction or increase in hepatic steatosis post-intervention). A total of 12 participants were classified as responders, and 7 were classified as non-responders (one participant was excluded due to missing MRI data at Week 8). Due to our small sample size, our subgroup analysis likely lacked sufficient power to detect true significant differences between groups. However, our research findings provide valuable insights into the understanding of pediatric MASLD despite these limitations. Please see Figure S6 for our subgroup analysis including power calculations.

As shown in Figure S6A, responders demonstrated a significant improvement in fasting glucose, LDL, plasma TG, TC, and hepatic steatosis from baseline to week 8 (*p* < 0.05), while no significant changes in these clinical traits were observed in non-responders. Both groups showed significant improvements in ALT, AST, GGT, BMI z-score, and DNL (*p* < 0.05) (Figure S6A). Additionally, while there were no significant differences in baseline clinical variables between responders and non-responders, we did find significant differences in the lipidome between groups. The baseline levels of total Cer and DG species, as well as the TG/DG ratio, were significantly lower in responders versus non-responders (DG: *p* = 0.040; Cer: *p* = 0.043, TG/DG: *p* = 0.041; Figure S7B). Moreover, in responders, the diet treatment led to a significant decrease in total TG and LPC species, as well as the TG/DG and PC/PE ratios (all *p* < 0.05), whereas these lipid classes remained unchanged in non-responders (Figure S7B).

We also investigated the difference in lipid co-expression networks between responders and non-responders. Among responders, Module 2 (M2 (blue), enriched in palmitate containing TG species); M8 (magenta; LPC and DG species); and M9 (pink; PC and ChE species) all significantly changed with the diet treatment (*p* < 0.05). However, these same modules were not significantly changed in non-responders (Figure S6B). M4 (yellow: enriched in long-chain SM and HexCer) was significantly increased in both responders and non-responders with the diet treatment. Moreover, there was a significant difference in baseline values of M7 (black; enriched in DG) and M11 (purple; enriched in Cer) between groups. The LSEA results, depicted in Figure S6C, illustrate that both responders and non-responders exhibited a significant decrease in the TG lipid class. However, responders showed a more pronounced NES compared to non-responders. The SM lipid class was increased in both responders and non-responders, with responders again displaying a more pronounced change. On the other hand, Hexcer, DG, and Cer lipid classes demonstrated contrasting trends in responders versus non-responders. However, the change in Cer and HexCer reached statistical significance only in responders (*p* < 0.05). Furthermore, the LSEA revealed that modules rich in TG, such as M2 (blue), M1 (turquoise), M10 (greenyellow), and M12 (tan), were reduced in both responders and non-responders (Figure S6D). Of note, only M2 (blue) achieved statistical significance in both groups (responders: *p* = 3.2 × 10^−4^, non-responders: *p* = 4.4 × 10^−3^). Additionally, M4 (yellow), enriched in SM, significantly increased in both groups (responders: *p* = 4.8 × 10^−3^ non-responders: *p* = 3.2 × 10^−3^). 

Integrating the WGCNA results with the clinical traits, the network among responders revealed that the TG-enriched M2 (blue; primarily containing palmitate (16:0) fatty acyl chains) exerted the strongest influence on select clinical traits compared to other modules based on the eigenvector centrality score. Additionally, M2 (blue) showed significant positive correlations with hepatic steatosis (cor = 0.59, *p* = 0.045) and DNL (cor = 0.71, *p* = 0.022), and significant negative correlations with SM-enriched modules (M3 (brown): cor = −0.61, *p* = 0.034). Interestingly, M2 (blue) did not play a significant role in the non-responder network. Additionally, in the responder network, fasting insulin demonstrated significant correlations with DG and LPC-enriched modules (M7 (black), M8 (magenta), and M6 (red)), while in the non-responder network, fasting insulin correlated with DNL and the ether-TG-enriched M12 (tan). Furthermore, M10 (green-yellow), containing a higher number of carbons and relatively unsaturated TG species, correlated directly with plasma glucose levels in non-responders. Figure S6E illustrates correlations in each network >0.7. Please see Supplementary Tables S14 and S15 for all correlations, 95% confidence intervals, and *p*-values. 

## 4. Discussion

We investigated systemic lipidome changes associated with the provision of a LFSD and subsequent regression in hepatic steatosis among adolescent boys with MASLD. In the dietary intervention group, there were significant changes in the lipid profile of participants, specifically reduced levels of TGs, DGs, ChE, PC, and LPC species. Prior research typically has found these same lipids elevated in MASLD progression; therefore, our findings may indicate regression and normalization of key biological processes, including lipid metabolism, insulin resistance, and lipotoxicity. Additionally, our targeted oxylipins assay revealed novel changes in eicosanoid species, suggesting improvements in oxidative stress with the diet treatment. Interestingly, our DE analysis revealed that several individual lipid species associated with MASLD were elevated after the diet treatment, indicating that pathogenic plasma lipidome characteristics persist. This is evident because some participants responded favorably to the diet treatment while others did not. Therefore, a longer intervention period may be warranted in future studies.

### 4.1. Changes in Lipid Classes and Individual Lipid Features with the LFSD Treatment

MASLD is driven by the accumulation of plasma and intrahepatic TG due to factors such as enhanced DNL, impaired beta-oxidation, and reduced VLDL secretion [61,62]. DGs also play a role in MASLD progression, as they can disrupt insulin signaling, promoting hepatic insulin resistance and inflammation [63,64,65,66,67]. Moreover, a stepwise increase in the TG/DG ratio has been observed in adults, progressing from those with healthy livers to those with MASLD and MASH [62]. Our findings, which revealed a reduction in total TGs, DGs, and the TG/DG ratio following the LFSD intervention, coupled with a decrease in hepatic steatosis, suggests a potential association between these changes with improved lipid metabolism and insulin resistance. Moreover, these findings may point to the early stages of MASLD regression [63,64,65,66,67].

In line with our previous metabolomics study [25], we observed a change in glycerophospholipids following the dietary intervention, including a significant decrease in the abundance of PC and LPC lipid classes. Perturbations in glycerophospholipid metabolism have been implicated in MASLD progression [68]. More specifically, PC species, a major component of cell membranes, have been observed at higher circulating levels in MASLD and MASH patients compared to healthy individuals [69]. LPC species, originating from PC hydrolysis in the lipid bilayer, have been associated with hepatic lipotoxicity by inducing endoplasmic reticulum (ER) stress, mitochondrial dysfunction, and hepatocyte apoptosis [29,37,70]. Notably, pediatric studies have revealed a direct association between LPC and MASLD severity [36]. In our study, the decline in total LPC was primarily driven by a decrease in the abundance of specific species, including LPC (16:0), LPC (18:0), LPC (18:1), and LPC (18:2), which are known to stimulate TG accumulation in hepatocytes [71]. These lipid species have also been observed to decrease with weight loss among patients with obesity [72]. Therefore, the post-intervention declines in both PC and LPC levels may relate to the reduction in hepatic steatosis and suggests improvements in lipotoxicity.

MASLD has also been characterized by free cholesterol accumulation without a corresponding increase in ChE [73]. However, a recent lipidomics study in adults highlighted that ChE, among other plasma lipid classes, helped distinguish those with healthy livers from those with steatosis and MASH [74]. The liver is the major organ for cholesterol metabolism [75]. Free cholesterol can be esterified within hepatocytes to form ChE for storage in lipid droplets or excretion as part of VLDL [76]. Following the dietary intervention, we observed a significant decrease in total ChE. This observation suggests that the LFSD and reduction in hepatic steatosis are linked with improved cholesterol metabolism.

Although total SM and Cer were nonsignificantly decreased with the LFSD, several individual SM and Cer species were significantly changed in the intervention group. The PLS-DA results revealed that some of the top discriminatory features between baseline and Week 8 included SM and Cer species. Previous studies have shown increased SM and Cer concentrations in both steatosis and MASH [77]. The metabolism of SM produces Cer, which function as signaling molecules with physiological effects that contribute to MASLD progression [30]. More specifically, increased plasma Cer concentrations are known to promote insulin resistance and increase the production of reactive oxygen species (ROS), contributing to inflammation and hepatocyte apoptosis [78,79,80,81]. Other studies suggest that the concentrations of SM and Cer species vary in conditions of metabolic dysfunction due to various factors such as age, gender, duration of dietary intervention, and disease severity [82,83,84]. Interestingly, Cer(d18:1_25:1) increased after the LFSD and was negatively correlated with both hepatic steatosis and DNL. Although little is known about the role of Cer(d18:1_25:1) in human metabolism, in general, the length and degree of saturation of the sphingosine backbone and the amide-linked acyl chain impacts the physiological function of sphingolipids, including Cer [85]. Very-long-chain Cer, such as C24 and C25, have been shown to play a protective role against the development of insulin resistance and steatosis, while saturated Cer with shorter chain lengths, such as C16, have been associated with hepatic TG accumulation [86]. Therefore, the very-long-chain, unsaturated Cer(d18:1_25:1) may offer therapeutic benefits for patients with MASLD. However, further studies are needed to fully understand and verify its use in treatment.

Serine plays a significant role in sphingolipid synthesis through palmitoyl-CoA condensation [87], and serine deprivation has been shown to promote a reduction in sphingolipid species [88]. Additionally, serine supplementation has prevented the onset of steatosis in animal studies [89]. Interestingly, in our previous metabolomics study of the same study participants [25], elevated plasma serine levels occurred post-intervention. Therefore, the observed increase in serine may have contributed to the rise in individual Cer and SM species in our present study.

Overall, the significant changes in lipid classes support our hypothesis that the LFSD treatment improves lipid metabolism, consistent with prevailing literature on MASLD pathophysiology. However, contrary to our expectations, several individual lipid species associated with MASLD progression, namely SM, Cer, TG, and LPC species, were elevated after the diet treatment. Notably, while the intervention group demonstrated a marked reduction in hepatic steatosis overall, only one participant had hepatic steatosis levels below 5% after the 8-week duration. We speculate that the increase in individual lipid species linked with MASLD stems from the lasting effects of the disease. Supporting this notion, a recent metabolomics study analyzed participants from the PREDIMED (Prevention of Disease with Mediterranean Diet) trial [90]. It showed that participants who demonstrated MASLD reversion over a 3.8-y follow-up experienced only minimal changes in lipid species. Notably, despite anticipated improvements in lipotoxicity, there were only slight decreases in SFA, PE, and LPC species. The authors concluded that some of the side effects of MASLD in participant metabolic profiles may persist. Therefore, further research is needed to understand the mechanisms of intrahepatic lipid remodeling in MASLD patients undergoing dietary treatment.

### 4.2. Lipid Co-Expression Network Analysis

Our lipid co-expression network analysis provided further biological insights into the changes in lipid metabolism that occurred in the intervention group. The lipid modules that significantly changed with the diet treatment, including Module 2 (M2 (blue); enriched in palmitate (16:0) containing TG), M9 (pink; PC species), and M4 (yellow; SM and HexCer species), were significantly correlated with hepatic steatosis. More specifically, M2 (blue) was also positively correlated with DNL and decreased with the diet treatment. Since palmitate is the initial fatty acid synthesized during DNL [91], this finding provides additional evidence that the LFSD improves both hepatic steatosis and DNL. Additionally, the top three hub lipids in M9 (pink), which increased with the diet treatment, were PC (16:0_20:4), PC (18:0_20:4), and PC (38:6). As mentioned above, an increase in the total content of PC has been observed in MASLD and MASH. However, other studies have shown that individual phospholipid species, such as PC (38:6), show a stepwise decrease with MASLD progression [92]. Therefore, these individual hub PC species may be indicative of MASLD improvement. Furthermore, in M4 (yellow), which also increased with the diet treatment, the top three hub lipids were very-long-chain SM species (SM(d40:1) and SM(d43:1)) and a very-long-chain HexCer (Hex2Cer(d18:1_24:1)) species. A recent study in mice revealed that very-long-chain sphingolipid species exhibit a protective role against the development of glucose intolerance and hepatic insulin resistance [93]. Therefore, since M4 (yellow) was also correlated with hepatic steatosis, DNL, and BMI z-scores, this may relate to improvements in insulin sensitivity with the diet treatment.

Other lipid modules from the WGCNA did not significantly change with the diet treatment. However, network analysis revealed interesting insights into their connections with clinical traits. For example, M12 (tan), enriched in ether-TGs with a fatty acyl chain length of C16 or C18, was highly associated with DNL. DNL and ether lipid synthesis are closely linked processes, with acetyl-CoA playing a pivotal role as a precursor in each pathway. In this context, fatty acid synthase (FASN) utilizes acetyl-CoA to synthesize palmitate, a key step in DNL [94]. Interestingly, research has demonstrated that loss of FASN impairs DNL and concurrently diminishes ether lipid formation [77]. This interrelation suggests a shared pathway and underscores the association between the biosynthesis of ether lipids and DNL. Additionally, M7 (black), primarily composed of DG with a chain length of C16 and C18, showed the highest positive association with plasma TG levels. TG species are hydrolyzed to DG and fatty acids. Tracing studies of the triglyceride cycle in human plasma suggest that the hydrolysis of TG to DG might better reflect total plasma TG levels, providing evidence for this association [95]. M10 (green-yellow), which primarily contained shorter chain saturated and monounsaturated TG species, strongly correlated with fasting glucose and TG levels. Aligning with this observation, TG species with relatively low carbon numbers and double bonds are known to correlate positively with HOMA-IR [96]. Furthermore, both M6 (red) and M8 (magenta) were enriched in LPC species and were negatively correlated with fasting insulin levels. A recent in vitro study demonstrated that LPC may act as an effector of fatty acid-induced insulin resistance [97]. However, certain LPC species, with a higher carbon number and double bond content, such as LPC (22:6), which was found in M8 (magenta), and odd-chain saturated LPC species, such as LPC (17:0) in M6 (red), are linked with a decreased risk of type 2 diabetes [96,98].

### 4.3. Clinical and Lipidome Differences between Responders and Non-Responders to the LFSD

Precision nutrition has recently emerged as a field that aims to develop tailored nutrition interventions based on individual differences with the goal of improving health outcomes [99]. Research in adults has shown that MASLD is a heterogeneous condition due to differences in several factors, including genetic variants, lipid partitioning, body composition, and inflammation, which ultimately result in different responses to therapy [100]. To gain a deeper understanding of the variability in response to the LFSD, we stratified participants in the intervention group into two subtypes: responders (≥5% reduction in hepatic steatosis) and non-responders (<5% decrease or increase in hepatic steatosis) and examined differences in their lipidome and clinical traits. Although both responders and non-responders exhibited improvements in their lipid profiles and clinical variables, different trends emerged between groups at baseline and in response to the diet treatment.

While there were no statistically significant differences in baseline clinical variables between responders and non-responders, we did observe significant differences in the lipidome between groups. Notably, the responder subtype exhibited lower baseline levels of lipid species linked with lipotoxicity and MASLD progression, including Cer and DG species. In muscle tissue, DG species have been shown to impair insulin signaling, leading to insulin resistance [101]. As mentioned above, Cer species are similarly linked with impaired insulin sensitivity, oxidative stress, and inflammation [102]. Responders also presented with a lower TG/DG ratio associated with less severe disease [62]. Additionally, there was a significant decrease in the palmitate-enriched module (M2; blue) among responders, suggesting greater improvements in DNL. Therefore, the responder subtype was characterized by a less lipotoxic lipid profile at baseline, in contrast to non-responders who exhibited greater lipotoxicity, indicating greater disease severity. Thus, non-responders may require an extended diet treatment period or additional interventions to invoke a response. However, further analyses with a larger sample size are needed to substantiate our preliminary findings. Moreover, further research, including additional variables such as genetics, mitochondrial function, and the gut microbiome, would help to further elucidate the heterogeneity in response to a low-sugar diet in children with MASLD.

### 4.4. Changes in Oxylipin Species

Our targeted oxylipins assay revealed a significant decrease in 8-isoprostane, an eicosanoid in the prostaglandin family [103]. The plasma level of 8-isoprostane is considered a reliable marker of lipid peroxidation and oxidative stress in vivo [104,105,106]. A study in adults demonstrated that 8-isoprostane levels are significantly elevated in patients with MASLD and other chronic liver diseases compared to healthy controls [107]. Since we observed a significant decrease in 8-isoprostane, this suggests an improvement in lipid peroxidation and oxidative stress with the LFSD.

Another fate of arachidonic acid is the formation of epoxyeicosatrienoic acids (EETs) via the CYP epoxygenase/soluble epoxide hydrolase (sEH) pathway. This pathway is a central regulator of the hepatic inflammatory response [108]. EETs are known to have anti-inflammatory and anti-steatotic effects [109]. However, they are rapidly hydrolyzed to their corresponding soluble DiHETs, which are less biologically active [110,111]. Our study revealed a significant increase in 8,9 DiHET and a significant decrease in 14,15 DiHET following the diet treatment. A recent study in children with MASLD demonstrated that during the initial stages of hepatic steatosis, there is an increase in EETs and their corresponding DiHETs accompanied by increased CYP epoxygenase activity. However, as the disease progresses to fibrosis, there is a reduction in EETs and CYP epoxygenase activity [112]. Since our study showed both a significant increase and decrease in individual DiHET species, further investigation is needed to elucidate the effects of the LFSD on the CYP epoxygenase pathway.

### 4.5. Strengths and Limitations

Overall, this study provides further insights into which disturbances in lipid metabolism may be most responsive to reductions in dietary sugar and hepatic steatosis. Additionally, it is the first to examine the systemic biological changes, assessed by both untargeted and targeted lipidomics, linked to a therapeutic reduction in hepatic steatosis measured by MRI-PDFF in youth with MASLD.

The strengths of this study include the use of an optimized, high-resolution lipidomics approach and the repeated measures design, which allows for control of individual variation and increases confidence that the changes were due to diet intervention. Moreover, while it remains plausible that the alterations in the lipidome may have resulted from other factors, our confidence is bolstered by the absence of significant changes in dietary fat intake within the intervention group. This suggests that the observed changes are more likely a consequence of the reduction in dietary sugar and the improvement in hepatic steatosis.

Furthermore, our targeted oxylipins analysis allowed us to gain a deeper understanding of downstream changes in PUFAs such as arachidonic acid. Additionally, our subgroup analysis of responders and non-responders revealed further insight into the metabolic heterogeneity among participants in response to the LFSD. However, the limited sample size of our subgroup analysis was likely too small to reliably identify significant differences between the responders and non-responders. Further investigation utilizing a more extensive sample is essential to substantiate our preliminary observations. Another limitation of our research was our focus on Hispanic adolescent boys with MASLD, potentially making our results less applicable to broader pediatric demographics. Moreover, our total sample size may have lacked sufficient power, limiting our capacity to discern statistically significant variations related to the dietary intervention. Lastly, our in-depth evaluation of dietary fat intake was confined to subjects from Emory University and did not account for potential variances by study site.

## 5. Conclusions

Our lipidomics analysis offers insight into lipidome changes resulting from an LFSD and hepatic steatosis reduction among adolescent boys with MASLD. The alterations in the lipidome identified in the diet treatment group may represent regression biomarkers related to a decrease in hepatic steatosis and dietary sugar intake. Additionally, the reduction in palmitate-enriched TG species, the main byproduct of DNL, emphasizes the role of sugar reduction in suppressing DNL. Since DNL is a key driver of MASLD, this illustrates the importance of dietary strategies in managing this metabolic disease. Furthermore, the preliminary findings from our subgroup analysis revealed subtypes of pediatric MASLD, highlighting the need for tailored nutrition interventions in this patient population.

Looking forward, our findings lay a foundational step for additional research to validate our preliminary insights and expand our understanding of lipidome modifications in the context of MASLD management. Future studies, with a larger sample size and extended study period, are vital to corroborate our findings. Such studies will provide insight into how these alterations can be leveraged in developing therapeutic strategies for MASLD. Overall, our study underscores the critical role of nutritional interventions focused on reducing added sugar consumption to improve public health and prevent the onset and progression of MASLD among future generations.

## Figures and Tables

**Table 1:** Baseline characteristics of the sample population (n = 40).

Characteristic	Intervention, N = 20 ^1^	Control, N = 20 ^1^	*p*-Value ^2^
Age (y)	12.75 (1.80)	13.30 (1.81)	0.3
BMI (kg/m^2^)	33.70 (5.57)	32.29 (6.28)	0.4
BMI z-score	2.38 (0.28)	2.22 (0.48)	0.3
ALT (U/L)	132.30 (112.23)	91.90 (47.12)	0.4
AST (U/L)	65.30 (52.15)	49.85 (28.08)	0.7
GGT (U/L)	53.25 (34.17)	52.30 (35.00)	>0.9
Glucose (mg/dL)	91.05 (9.80)	90.45 (13.85)	>0.9
Insulin (uIU/L)	42.99 (22.99)	53.62 (43.76)	0.5
HOMA-IR	9.94 (5.99)	12.76 (12.67)	0.6
TG (mg/dL)	144.15 (83.01)	148.00 (48.67)	0.5
TC (mg/dL)	162.10 (42.46)	157.25 (31.30)	0.9
LDL (mg/dL)	100.50 (34.85)	95.20 (24.06)	0.7
HDL (mg/dL)	40.10 (7.06)	40.50 (7.16)	>0.9
Hepatic steatosis (%)	24.93 (11.21)	20.78 (8.16)	0.2

^1^ Mean (SD), ^2^ Kruskal–Wallis rank sum test. Participant baseline characteristics are expressed as mean (standard deviation (SD)) for continuous variables. Kruskal–Wallis rank sum test was used to compare the dietary intervention and control groups. Statistical significance was considered as *p* < 0.05. HOMA-IR was calculated as fasting glucose (mg/dL) × fasting insulin (uIU/L)/405. Abbreviations: ALT, alanine aminotransferase; AST, aspartate aminotransferase; BMI, body mass index; GGT, gamma glutamyl transferase; HDL, high density lipoprotein cholesterol; HOMA-IR, homeostasis model assessment of insulin resistance; LDL, low density lipoprotein cholesterol; TC, total cholesterol; TG, triglycerides.

## Data Availability

The raw data supporting the conclusions of this article will be made available by the authors on request.

## Supplementary Materials

The following supporting information can be downloaded at: https://www.mdpi.com/article/10.3390/metabo14040191/s1, Figure S1. Untargeted lipidomics analysis findings in the dietary intervention and control group, Figure S2. Repeated measures correlations between the top three individual lipid species with hepatic steatosis and DNL, Figure S3. WGCNA results, Figure S4. Additional WGCNA results, Figure S5. Oxylipins analysis findings, Figure S6. Differences in lipids and clinical traits between responders and non-responders to the LFSD treatment, Figure S7. Differences between responder and non-responder sub-types of MASLD. Table S1. Untargeted chromatography parameters, Table S2. Untargeted instrumental parameters, Table S3. LipidSearch output, Table S4a. Oxylipins chromatography gradient, Table S4b. Endocannabinoids chromatography gradient, Table S5. Targeted mass spectrometry instrumental parameters, Table S6. Oxylipins transition list, Table S7. Baseline and Week 8 Dietary Fat Intake from a sub-sample of 20 participants, Table S8. Differential expression analysis results, Table S9. Repeated measures correlations between individual lipid species & hepatic steatosis, Table S10. Repeated measures correlations between individual lipid species & DNL, Table S11. WGCNA results, Table S12. Eigenvalues (ME) of each sample, Table S13. Correlations between WGCNA modules and clinical traits in the dietary intervention group, Table S14. Correlations between WGCNA modules and clinical traits in the responder subgroup, and Table S15. Correlations between WGCNA modules and clinical traits in the non-responder subgroup.
