# Supplementary material for: Lipidome Changes Associated with a Diet-Induced Reduction in Hepatic Fat among Adolescent Boys with Metabolic Dysfunction-Associated Steatotic Liver Disease
Source: Metabolites. 2024 Mar 28;14(4):191. doi: 10.3390/metabo14040191 (PMC11052520; doi:10.3390/metabo14040191)
Supplement: Supplementary file 1 [file metabolites-14-00191-s001.zip › Supplementary information_revised_FINAL_01.pdf]

Figure S1. Untargeted lipidomics analysis findings in the dietary intervention and control group.

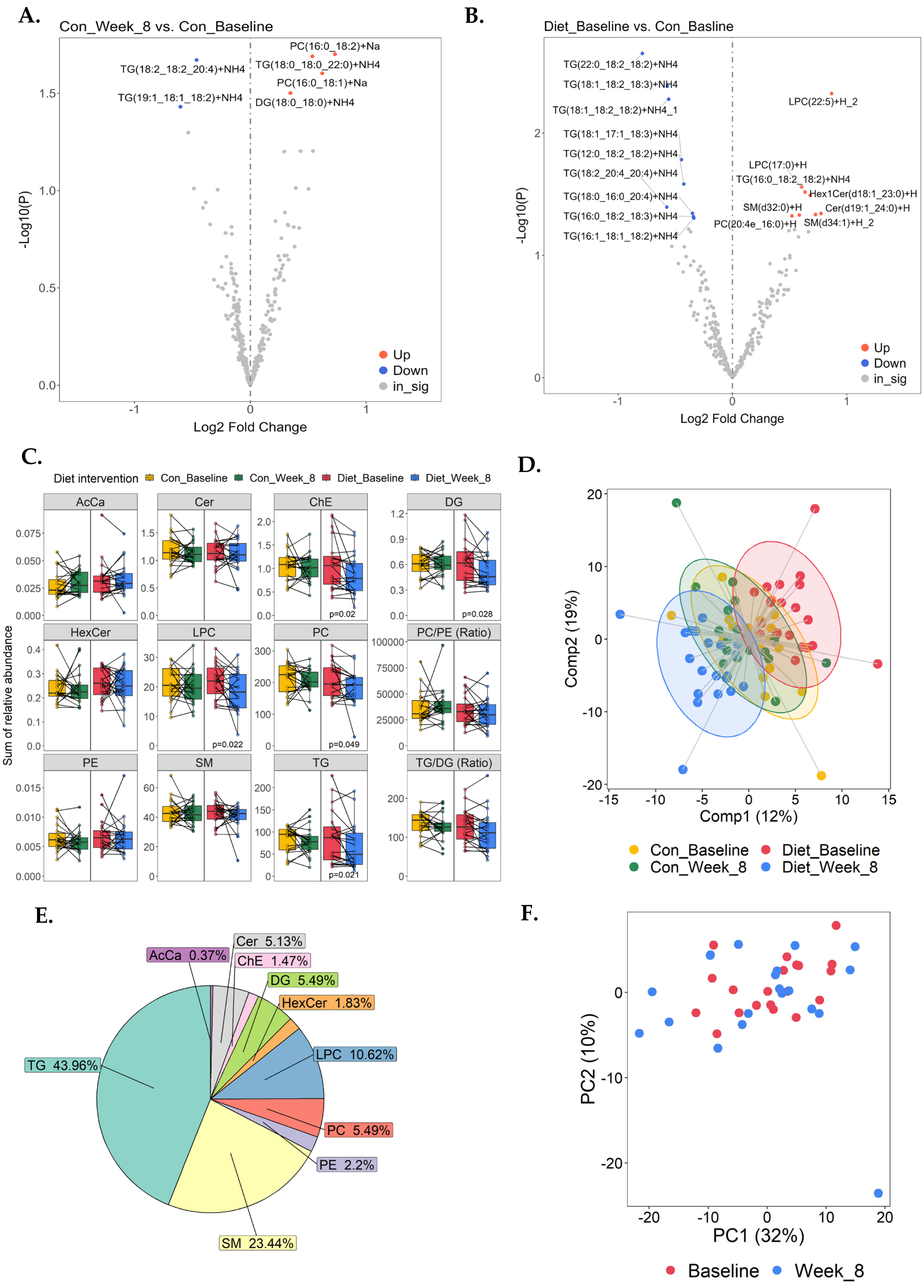

(A) Volcano plot illustrating the log_2 fold change versus $-log_{10}$(p-value) for pairwise comparisons, as calculated by the R package limma. Lipid species with statistical significance ($p<0.05$) with a significant difference of means between the control group at baseline (Con_Baseline) compared to week 8 (Con_Week_8) are labelled. **(B)** Volcano plots illustrating lipid species with statistical significance ($p<0.05$) of difference of means between the control group (Con_Baseline) and diet treatment group (Diet_Baseline) at baseline. **(C)** Boxplots showing the difference in relative abundance of lipid classes between baseline and week 8 for the control and diet treatment groups. P-values achieving statistical significance ($p<0.05$) are presented in the figure. The points were highlighted as red for increased fold changes and blue for decreased fold changes after dietary intervention. Red and blue represent pre-(Baseline or Diet_Baseline) and post-dietary intervention (Week_8 or Diet_Week_8), respectively. Yellow and green represent control group with pre-(Con_Baseline) and post-dietary intervention (Con_Week_8), respectively. **(D)** Supervised PLS-DA analysis of lipidomic data with the first two components (Comp 1 and Comp 2) are presented and 95% confidence ellipses are drawn surrounding the cluster core for the control and diet treatment groups. **(E)** Pie chart illustrating the plasma lipid composition as a percentage of the total number of lipid species from the untargeted lipidomic analysis for all 10 lipid classes measured **(F)** PCA of lipidomic data for the diet treatment group with PC1 and PC2 on the x and y-axis respectively.

Figure S2. Repeated measures correlations between the top three individual lipid species with hepatic steatosis (A) and DNL (B) among participants in the diet treatment group (n=16-19*).

A.

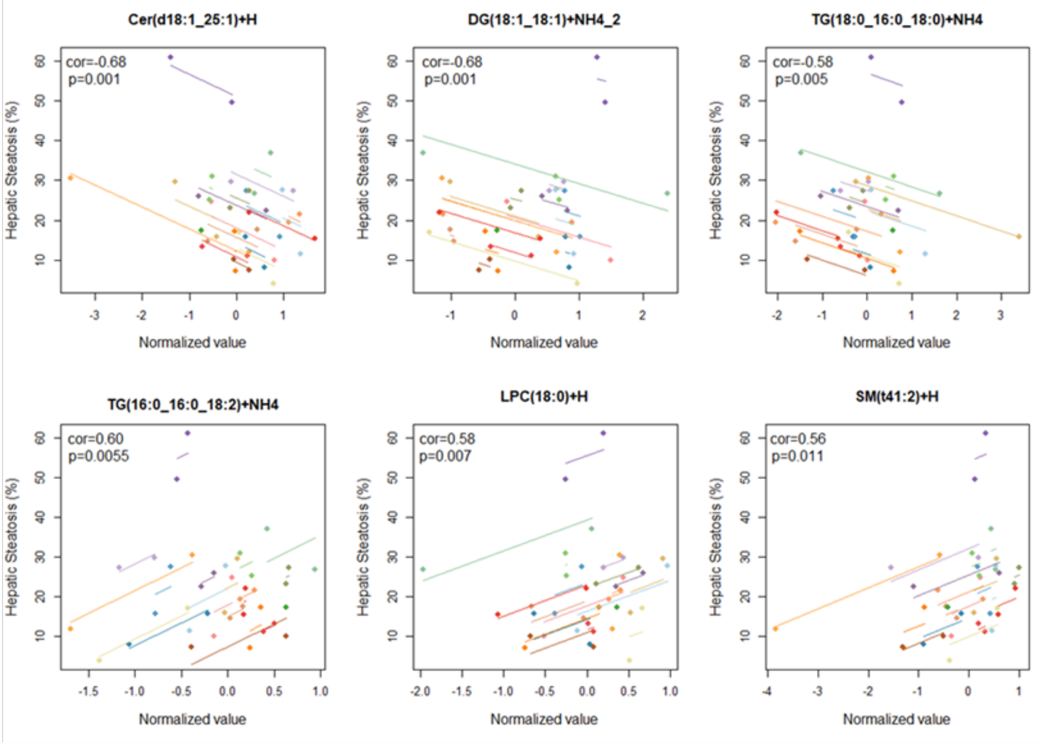

B.

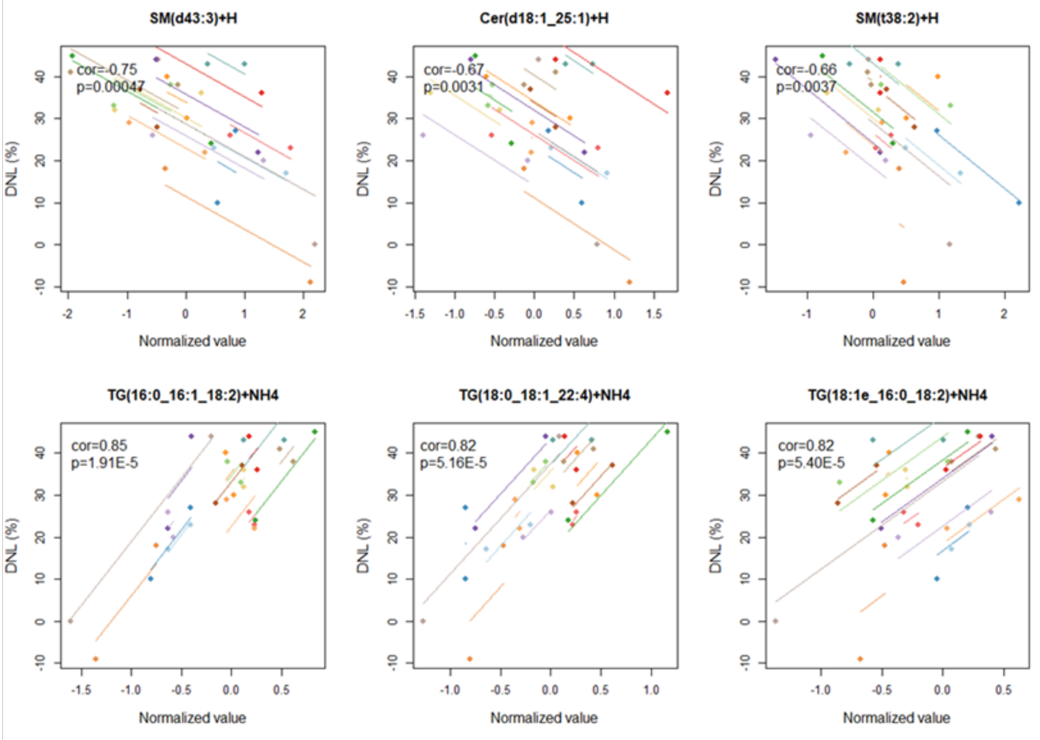

(A) The top three correlations between lipid species and hepatic steatosis. **(B)** The top three correlations between lipid species and DNL. The top panel of Figures A and B presents the top three lipids with negative correlations, and the bottom panel presents the top three lipids with positive correlations. Each individual subject's data and corresponding lines from the paired analysis are shown in a different color. Rmcorr coefficients (cor) and corresponding p values (p) are presented in each plot.

Figure S3. WGCNA results.

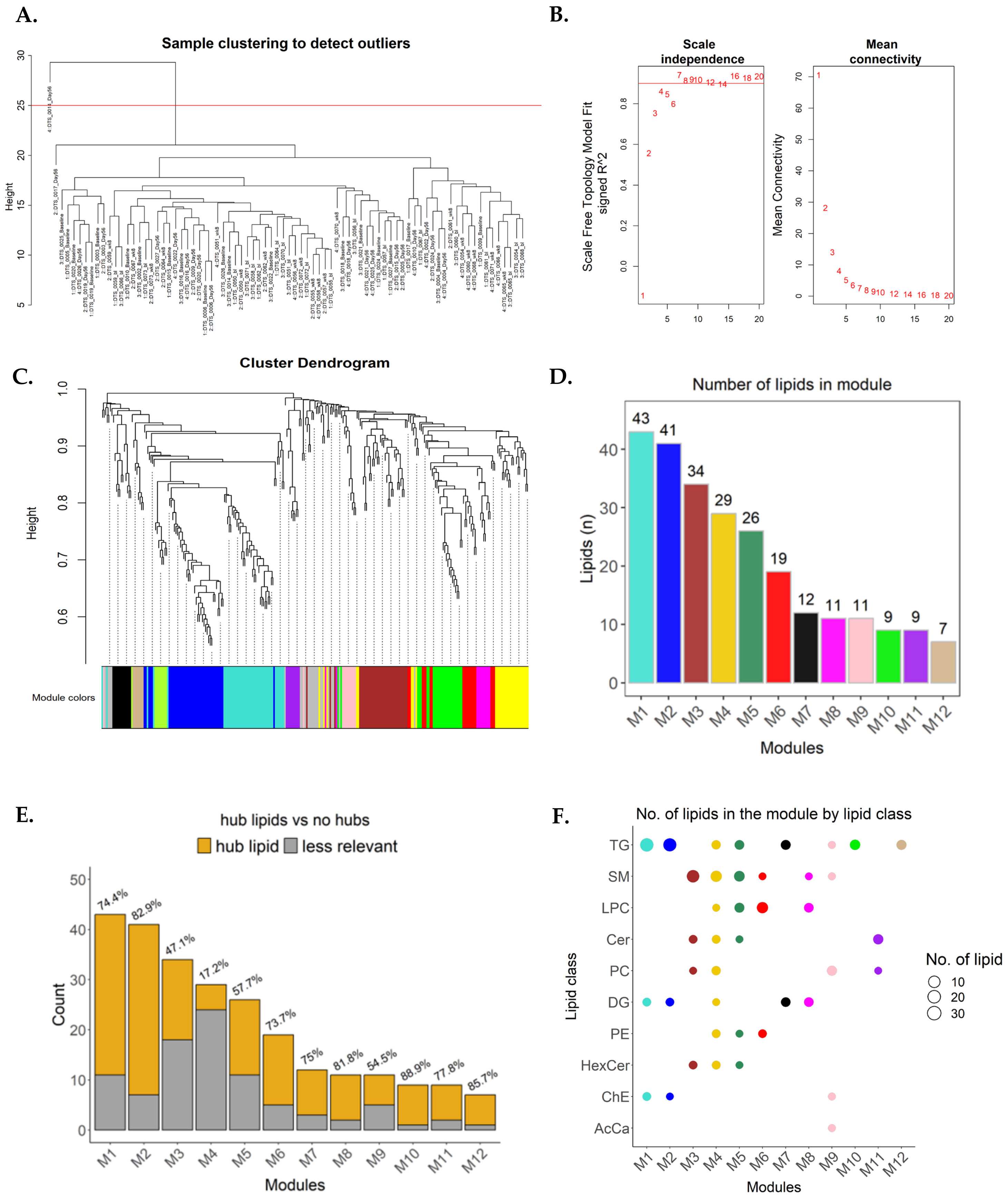

(A) Sample tree illustrating outliers. One sample was over the cutoff threshold of 25 and was removed from analysis. **(B)** Scale-free fit index for various soft-thresholding powers (β) (left). When the soft power threshold is 7, R^2 reaches the highest value (R^2: 0.942). The red line represents the merging threshold (0.9). Analysis of the mean connectivity for various soft-thresholding powers (right). In all, 7 was the best-fit power value with mean connectivity <3 (2.590) **(C)** Module cluster dendrogram illustrating the distribution of lipids in each module. To enhance readability, the colors of gold2, seagreen, and green2 were employed as replacements for the colors of yellow, green, greenyellow modules, respectively. **(D)** Bar chart showing the number of lipids in each module. **(E)** Stacked bar chart demonstrating the percentage of hub lipids in each module with a MM >0.65. **(F)** Bubble plot showing the overall lipid composition in each module. The size of the bubble indicates the number of lipids in each lipid class as indicated in the legend.

Figure S4. Additional WGCNA results.

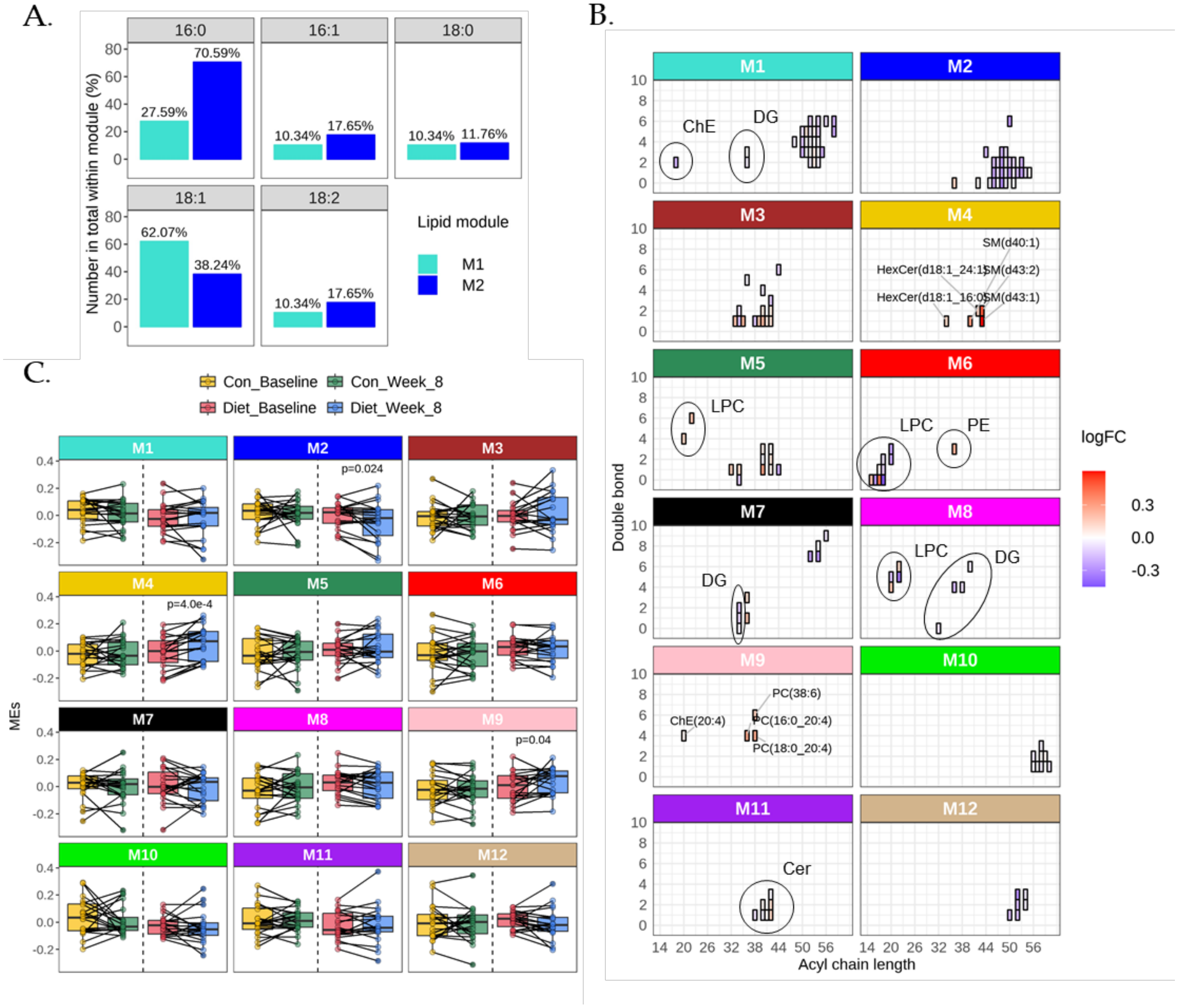

(A) Bar charts illustrating the comparison of acyl fatty acid chains in TG species between blue and turquoise modules. The percentage was determined by dividing the count of the indicated acyl fatty acids shown in an individual TG species by the total TG species per module, then multiplying by 100. **(B)** Heatmaps showing the log fold changes of Diet_Week_8 vs. Diet_Baseline for each module. The hub lipid species in each module shown in the plots are based on their corresponding double bonds and acyl chain length in the structure. The modules black, blue, turquoise, greenyellow, and tan primarily contain TG species unless otherwise specified. The green and brown modules mainly contain SM species unless otherwise specified. **(C)** Boxplots showing the difference in ME values between baseline and week 8 for the control and diet treatment groups. P-values achieving statistical significance (p<0.05) are presented in the figure.

Figure S5. Oxylipins analysis findings.

A.

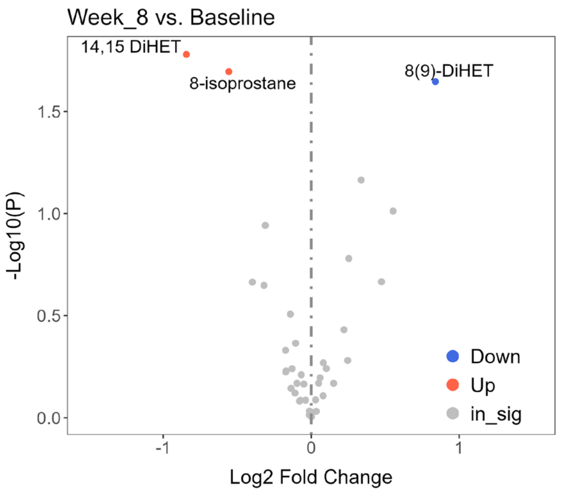

B.

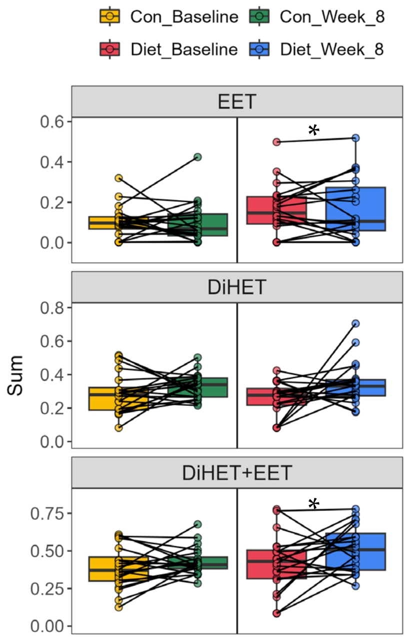

(A) Volcano plot showing the $\log_2$ fold change versus the $-\log_{10}$(p-value) for pairwise comparisons. Oxylipin species that significantly increased in abundance are shown in red, while those that significantly decreased are indicated in blue. **(B)** Boxplots showing the change in total DiHETs (sum of 8,9-DiHET, 10,11-DiHET, and 14,15-DiHET), total EETs (sum of 8,9-EET, 10,11-EET, and 14,15-EET), as well as the sum of total DiHET and EET species from baseline to week 8 in the intervention and control groups.

Figure S6. Differences in lipids and clinical traits between responders (R) and non-responders (NR) to the LFSD treatment (R: n=12; NR, n=7).

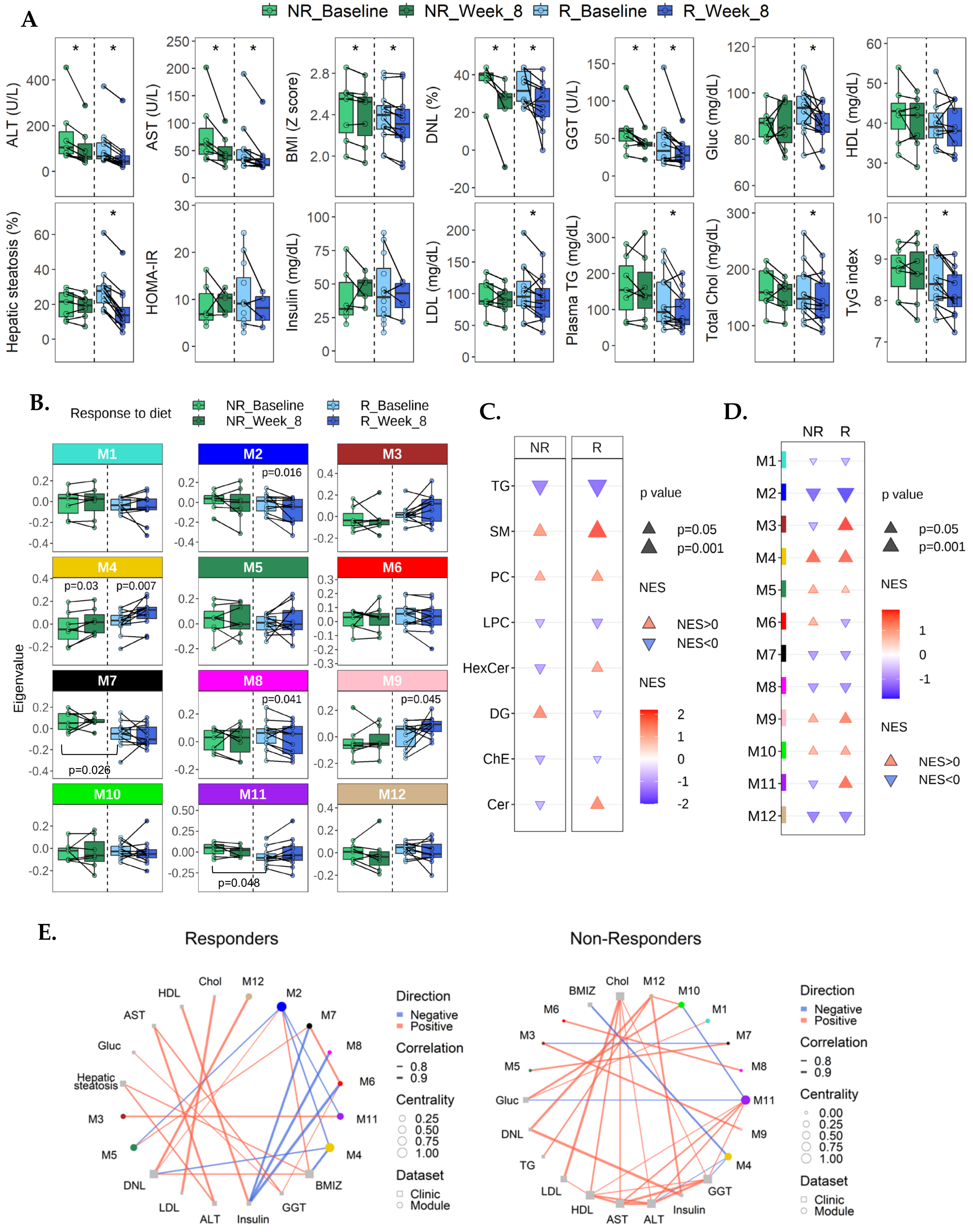

(A) Boxplots illustrating the change in clinical variables from baseline to week 8 among R and NR. Statistical significance ($p<0.05$, **power=0.49 based on a one-sided paired t-test**) is indicated using an asterisk. **(B)** Boxplots illustrating the difference in abundance of lipids from baseline to week 8 for each lipid module between R and NR. The p-values are listed for modules with statistically significant differences ($p<0.05$, **power=0.276 based on a one-sided t-test with an unequal sample size).** Black horizontal bars with the p-value underneath are used to indicate modules with significant differences between R and NR at baseline. **(C)** A bubble plot illustrating the differentially enriched lipid classes **within each subgroup**. **(D)** A bubble plot illustrating the differentially enriched lipid modules **within each subgroup** based on the WGCNA results. The normalized enrichment scores (NES), **based on the paired within-group fold changes,** are presented using a color scale from -1 (blue) to 1 (red). The size of the triangle represents the p-value (the larger the triangle, the smaller the p-value). **(E)** Responder and non-responder networks illustrating correlations **>0.7** and eigenvector centrality scores between the WGCNA modules and clinical traits. Correlations were calculated using the repeated measures correlation method, and statistical significance was set at $p<0.05$ **(in the responder subgroup, at a power of 0.8, the cor is a minimum of 0.72, and in the non-responder subgroup, at a power of 0.8, the cor is a minimum of 0.87).** Connecting lines are color-coded, with red indicating positive correlations and blue indicating negative correlations. The size of the nodes (circles and squares) for each module indicated the eigenvector centrality score (a larger circle/square indicates a higher score). **The statistical power was calculated using the pwr R package.**

Figure S7. Differences between responder and non-responder sub-types of MASLD.

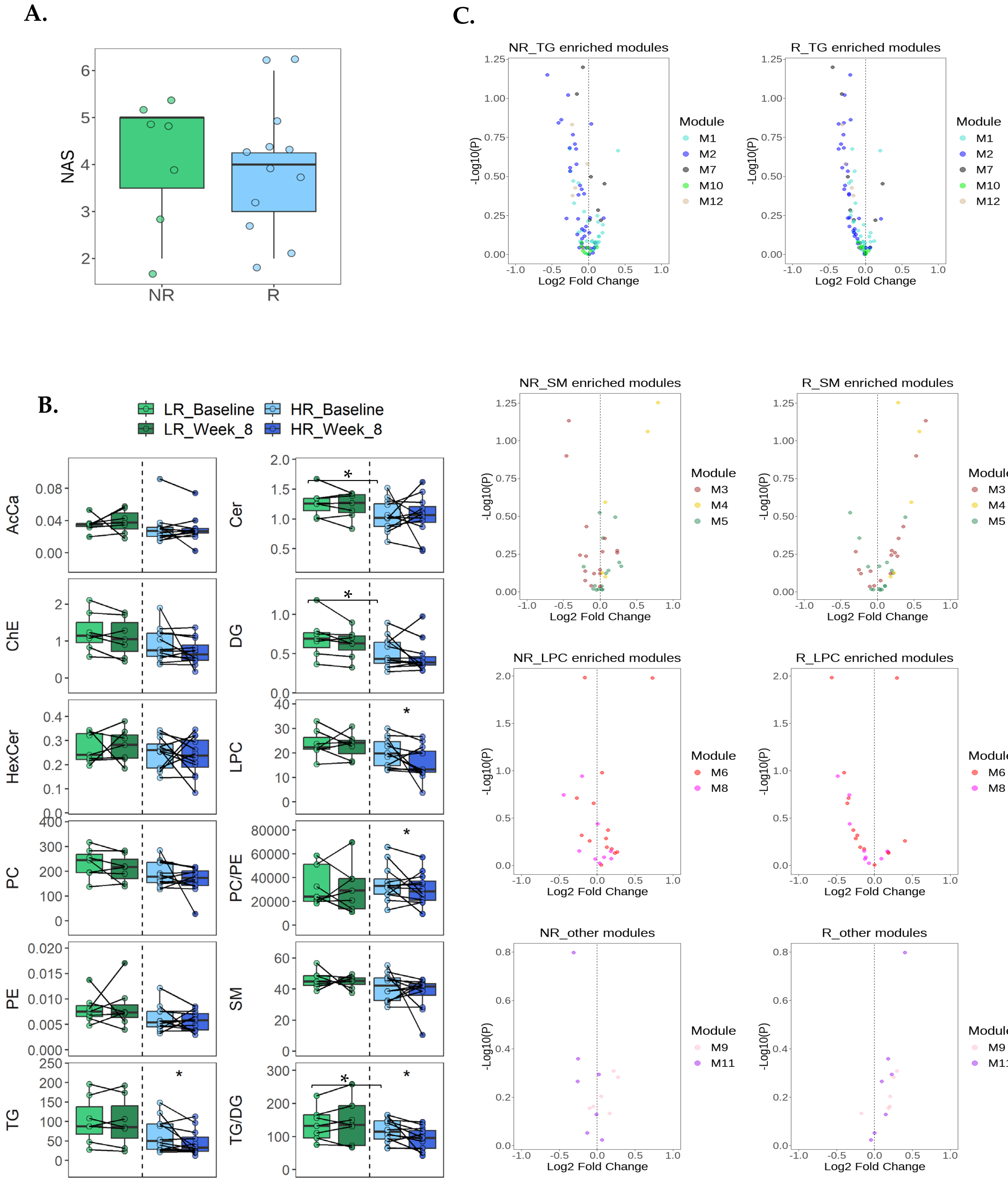

(A) Boxplots showing the NAS values at the baseline in Non-responders versus Responders. **(B)** Boxplots illustrating all matched paired measurements (baseline and week 8) for each lipid class between high responders (HR) and low responders (LR). A pairwise sample t-test was used to examine statistically significant differences between the means of the matched pairs within the group ($P<0.05$). A student t-test was utilized to assess statistical differences between the baselines of both HR and LR. Asterisks (*) indicate the statistical difference between groups ($P<0.05$). **(C)** Volcano plots showing the $\log_2$ fold changes versus $-\log_{10}$(p-value) for pairwise comparisons, as calculated by the R package limma. Based on the WGCNA results, the fold changes in HR and LR after dietary intervention were grouped by TG-enriched modules (black, blue, greenyellow, tan, and turquoise), SM-enriched modules (brown, green, and yellow), LPC-enriched modules (magenta and red), and other modules, such as the Cer-enriched purple module and PC-enriched pink module.
